# HIV viral suppression outcomes of Tenofovir- and Abacavir-based antiretroviral regimens among children on ART in Zambia

**DOI:** 10.3389/fpubh.2026.1860427

**Published:** 2026-07-08

**Authors:** Jonathan Gwasupika, Judie Magura, Lewis Phiri, Ephraim Chikwanda, Elizabeth Kabeta, Ray Handema

**Affiliations:** 1 National Health Research and Training Institute (NHRTI), Formerly Tropical Diseases Research Centre (TDRC), Ndola, Zambia; 2 Africa Health Research Institute (AHRI), Durban, South Africa; 3 Centre for Infectious Disease Research in Zambia (CIDRZ), Lusaka, Zambia; 4 School of Medicine, University College of Ndola, Ndola, Zambia

**Keywords:** adherence, antiretroviral therapy (ART), pediatric HIV, predicting factors, viral suppression

## Abstract

**Introduction:**

Use of ART has remarkably decreased HIV related morbidity and mortality rates globally. Children living with HIV in Zambia are initiated on an Abacavir-based regimen to suppress viral replication. This study assessed the achievement of HIV viral suppression among children taking Abacavir and Tenofovir-based regimens and the predicting factors associated with viral load suppression in Zambian children.

**Methods:**

This was a retrospective cross-sectional analysis of routinely collected data on all children diagnosed with HIV aged below 15 years attending the ART clinic from 1 January 2016 to 31 December 2018 at the Paediatric University Teaching Hospital in Lusaka, Zambia. Logistic regression was used to explore factors associated with HIV virological suppression. Ethical approval for the study was sought from the University of Zambia Biomedical Research Ethical Committee and the National Health Research Authority.

**Results:**

About 78.4% of children on ART achieved virological suppression (262/334). An increase in age showed about 20% reduced odds of virological suppression (AOR 0.80, CI 0.71–0.95), while a child on ABC/3TC/LPV-r combination of ART compared to TDF/XTC/DTG, showed about 88% reduced odds of virological suppression (AOR 0.12, CI 0.04–0.41).

**Conclusion:**

Despite an increased uptake of ART, the viral suppression found in this study was lower than the UNAIDS target of 95% of people on ART to be virally suppressed. Greater sensitization and education on the importance of treatment adherence are required to achieve this target, and further studies are needed to determine the factors contributing to the low viral load suppression in children.

## 1 Introduction

Despite the absence of a cure for Human immunodeficiency virus (HIV) infection (1), the introduction of antiretroviral therapy (ART) has substantially reduced HIV-related morbidity and mortality (2) and significantly improved patient survival (3). Globally, approximately 20.9 million people living with HIV were receiving ART by mid-2017 (4), including an estimated 1.8 million children, the majority of whom reside in Sub-Saharan Africa (5). In Zambia, where the HIV prevalence among children under 15 years was estimated at 1.1% (6), about 64% of children living with HIV had access to ART by 2017 (7).

HIV acquisition leads to progressive immune dysfunction (8), particularly through the depletion of CD4 + T cells (9). Therefore, to prevent immune suppression and uncontrolled viral replication (10), ART is initiated in children living with HIV as early as possible (11). Global Fast-Track targets call for 95% of those living with HIV to know their status, 95% of diagnosed individuals to receive sustained ART, and 90% of those on treatment to achieve durable viral suppression (12). However, these targets remain unmet in paediatric populations, where approximately 92.3% of children receiving antiretroviral therapy, only 54.3% achieve viral suppression loads, far below the 95% goal (13). Achieving and maintaining viral suppression in children remains challenging due to factors such as adherence difficulties, drug tolerability, and regimen selection (14).

In Zambia, children living with HIV are commonly initiated on Abacavir (ABC)-based regimens in combination with Dolutegravir (DTG), Lopinavir/ritonavir (LPV-r), or Efavirenz (EFV) (15). ABC has demonstrated safety and efficacy in children (16); however, data on virological outcomes remain limited (17). Moreover, predictors of suboptimal viral suppression in children receiving ART are not well documented (18). This study, therefore, aimed to determine the impact of ABC-and Tenofovir (TDF)- based regimens on viral load suppression and to identify factors associated with virological outcomes among children receiving ART at one of the children’s hospitals in Zambia.

## 2 Methods

### 2.1 Study design

The study used a retrospective cross-sectional design involving HIV-positive paediatric patients aged between 18 months and 14 years. Data were obtained through a review of medical records and extraction of routinely collected information from the national electronic medical record system, SmartCare. Relevant clinical and demographic variables were retrieved from the SmartCare database and the patient’s ART medical files.

### 2.2 Study site and population

The study was conducted among HIV-positive children receiving ART at the Paediatric Centre of Excellence (PCOE) within the University Teaching Hospital (UTH) in Lusaka, Zambia. The UTH Children’s Hospital is the largest specialized paediatric facility in the country and provides care to the highest number of children living with HIV nationally. The study included all children aged 18 months to 14 years who received ART services at PCOE between 1 January 2016 and 31 December 2018. Eligible participants were those initiated on Efavirenz (EFV) with either Abacavir (ABC) or Tenofovir (TDF), Lopinavir-ritonavir (LPV-r), or Dolutegravir (DTG), and had been on treatment for at least six months. Children without baseline CD4 count measurements or missing viral load results were excluded. A total of 334 children met the inclusion criteria and were included through complete enumeration of all eligible records within the study period.

### 2.3 Data collection tools and procedures

A semi-structured data abstraction tool (Supplementary Figure S1) was developed to extract relevant clinical and demographic information from the SmartCare electronic medical record system and patient ART files. The abstraction tool was tested to extract information for about 20 different children. The extracted information was later exported into STATA to assess for completeness and accuracy of data. The primary outcome variable was viral load measured atleast six months after ART initiation regardless of drug regimen. Virological suppression was defined as a plasma viral load of <1,000 copies/ml (19). Explanatory variables included age and sex of the child, duration on ART, weight, and baseline CD4 count (CD4 count at treatment initiation).

Data were independently entered by two trained data clerks, and a third clerk conducted verification for completeness and accuracy. Any incomplete abstraction forms were returned for correction unless missing information was not available in either the medical file or SmartCare, in which case a predefined code was assigned. Data were entered into Epi-Info software version 7 and subsequently exported to STATA version 16 for coding and statistical analysis.

### 2.4 Statistical analysis

Proportions were calculated for categorical variables, including sex of the child, viral load outcome (suppression or failure), CD4 count category, and ART regimen. For continuous variables that were non-normally distributed, the median and interquartile range (IQR) for age and duration of ART treatment were reported. Logistic regression analysis was performed to examine factors associated with virological suppression while adjusting for potential confounders. Odds ratios were used to report findings at univariate and multivariable analysis with a statistical significance level set at 5%, with 95% confidence intervals. All analyses were conducted using STATA version 16 SE (STATA Corp., College Station, Texas, USA).

### 2.5 Ethical considerations

Ethical approval for the study was obtained from the University of Zambia Biomedical Research Ethical Committee (Reference number 275-2019) and the National Health Research Authority. Permission to access patient records at the University Teaching Children’s Hospital and the Paediatric Centre of Excellence was granted. Confidentiality was strictly maintained by de-identifying all patients’ records using unique study identifiers. All files were securely stored in locked cabinets accessible only to authorized personnel. Since the study involved retrospective review of existing records with no direct patient contact, informed consent or assent was not required.

## 3 Results

**Figure 1:**
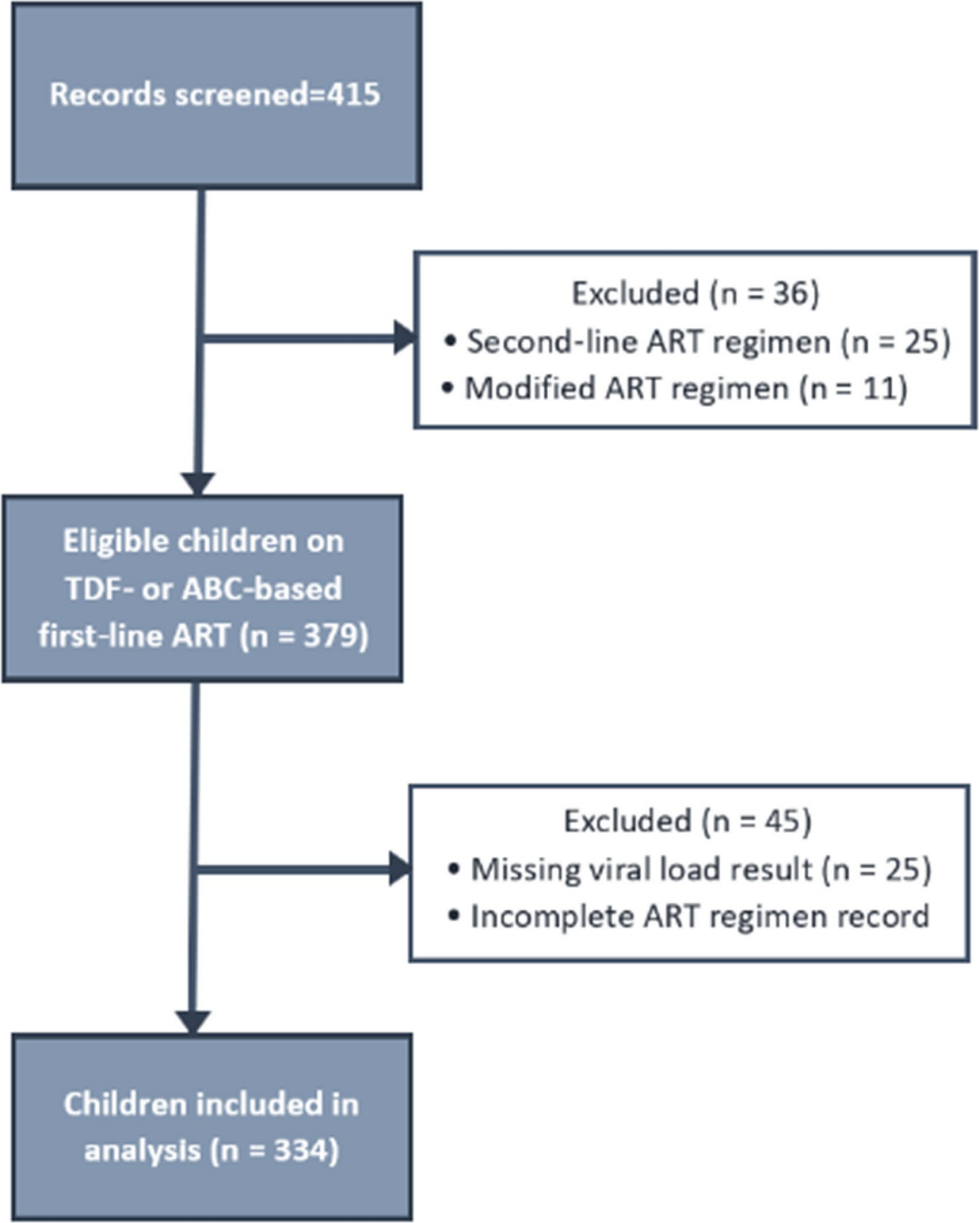
Flow diagram illustrating screening, exclusions, and final inclusion of children living with HIV receiving antiretroviral therapy in the analysis of viral suppression outcomes at the Paediatric Centre of Excellence, Lusaka, Zambia, from 1 January 2016 to 31 December 2018.

The study analyzed 415 available records of children living with HIV receiving ART care at the Paediatric Centre of Excellence, of which 334 participants met the inclusion criteria and were enrolled (Figure 1). The median age of children enrolled was 9 years (IQR: 5–11 years). There was a minimal difference in sex distribution, with 53.3% males and 46.7% females. Regarding ART regimens, 42.2% (141/334) of the children were receiving ABC/XTC/LPV-r, 29.3% (98/334) were on ABC/XTC/EFV, 18.3% (61/334) were on TDF/XTC/DTG, and 10.2% (34/334) were on TDF/XTC/EFV. Overall, 78.4% (262/334) achieved viral load suppression after at least six months of ART (Table 1).

**Table 1:** Demographic and clinical characteristics of HIV positive children seen at PCOE in Lusaka, Zambia, from 1st January 2016 to 31st December 2018, *N* = 334.

Variable	Frequency (%)
Age in years (Median, (IQR))^**^	9 (5 to 11)
Sex	
Male	178 (53.3)
Female	156 (46.7)
Duration on ART in weeks (Median (IQR)) ^**^	33 (20 to 49)
Time lag from Diagnosis to Treatment in weeks (Median (IQR)) ^**^	4 (1 to 54)
CD4 count	
<350cells	76 (22.8)
>350cells	258 (77.2)
Drug combination	
ABC/XTC/LPV-r	141 (42.2)
ABC/XTC/EFV	98 (29.3)
TDF/XTC/DTG	61 (18.3)
TDF/XTC/EFV	34 (10.2)
Viral load status	
< 1,000 copies/ml	262 (78.4)
> 1,000 copies/ml	72 (21.6)

IQR, interquartile range; ** Median and interquartile range reported; ABC, Abacavir; TDF, Tenofovir; XTC, Emtricitabine or Lamivudine; EFV, Efavirenz; DTG, dolutegravir; LPV-r, Lopinavir-ritonavir.

**Figure 2:**
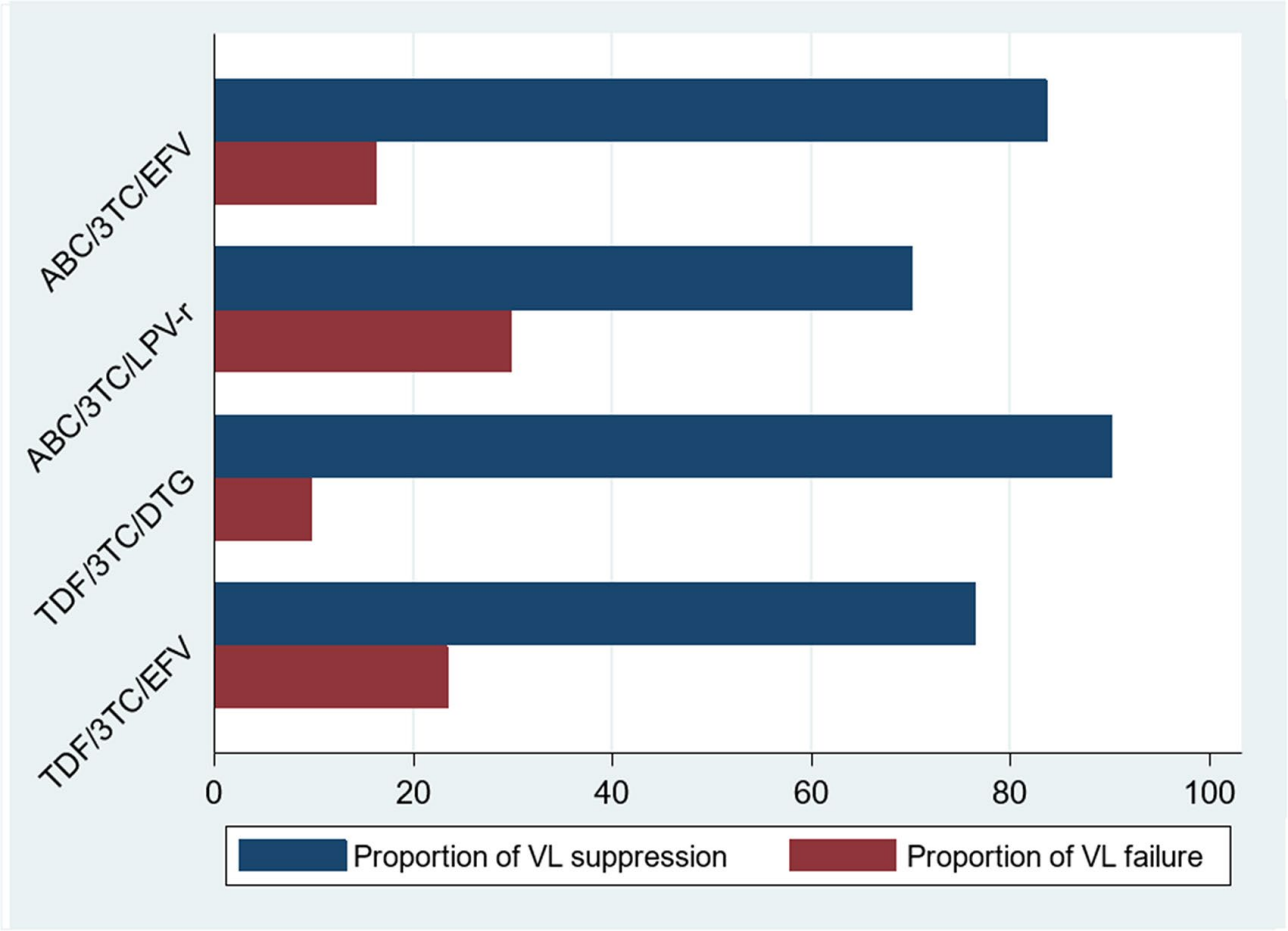
Proportion of virological suppression and virological failure among children living with HIV receiving antiretroviral therapy, by ART regimen, at the Paediatric Centre of Excellence, Lusaka, Zambia, from 1 January 2016 to 31 December 2018 (*N* = 334). Blue bars indicate virological suppression, and red bars indicate virological failure. Virological suppression was defined as a viral load of < 1,000 copies/ml measured after at least six months of ART.

**Figure 3:**
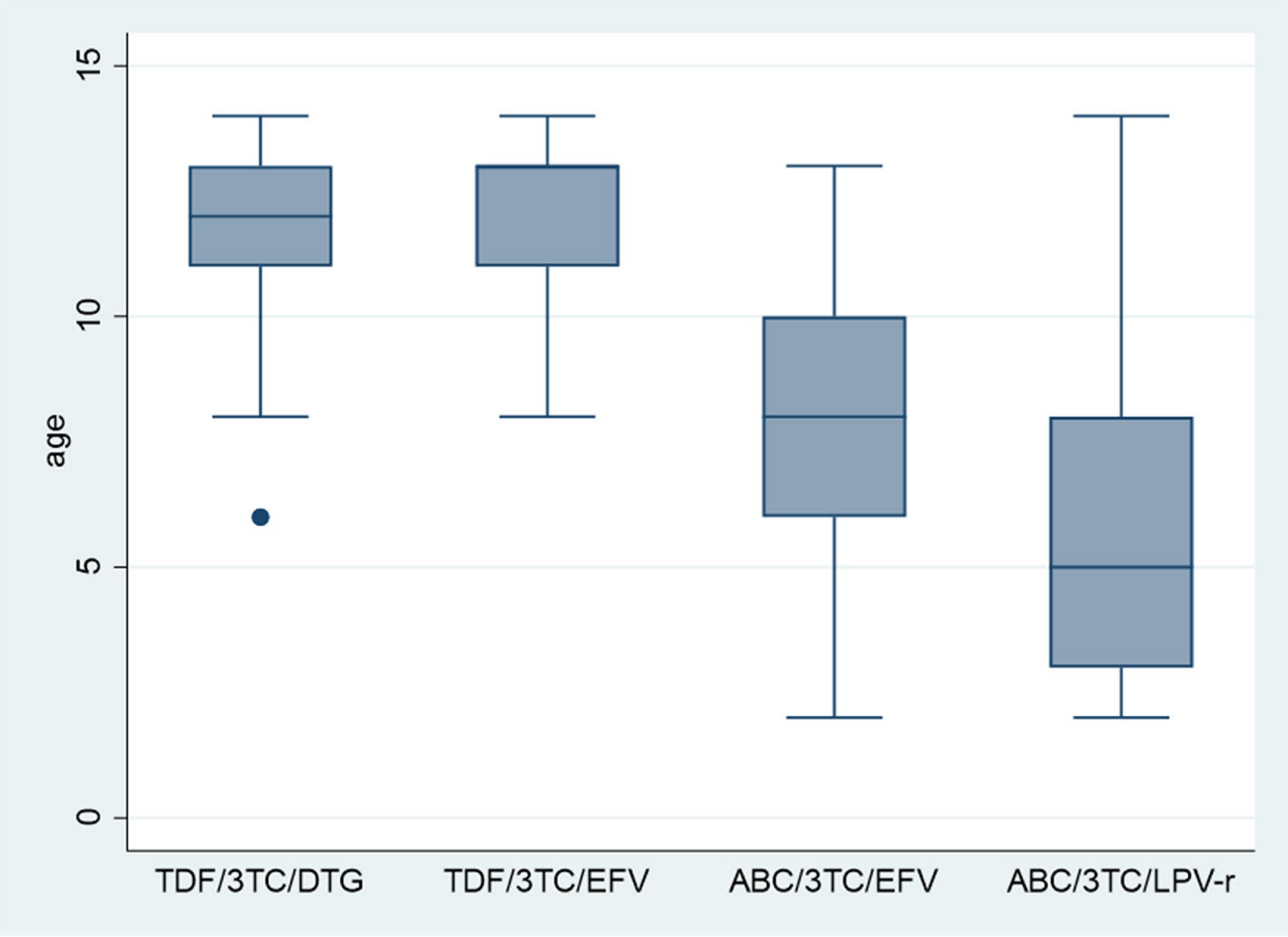
Distribution of age among children living with HIV receiving antiretroviral therapy, by ART regimen, at the Paediatric Centre of Excellence, Lusaka, Zambia, from 1 January 2016 to 31 December 2018 (*N* = 334). Boxes represent the median age and interquartile range, with whiskers indicating the distribution of ages within each regimen.

Children receiving TDF/3TC/DTG demonstrated the highest virological suppression rate (approximately 90%) and the lowest failure rate (about 10%). This was followed by children taking ABC/3TC/EFV with a suppression rate of 83%, TDF/3TC/EFV at 76%, while ABC/3TC/LPV-r showed the lowest suppression rate of 70% (Figure 2). Notably, children receiving TDF/3TC/DTG and TDF/3TC/EFV had a median age of over 10 years, whereas those on ABC/3TC/EFV had a median age between 5 and 10 years, and children on ABC/3TC/LPV-r had the youngest median age, at about 5 years (Figure 3). These observed differences in virological suppression and age distribution across ART regimens necessitated multivariable analysis to determine the independent predictors of viral load suppression.

The logistic regression analysis showed that age was an important predictor of virological suppression. Each additional year of age was associated with 20.0% reduction in the odds of achieving viral suppression (AOR = 0.80, 95% CI [0.71–0.95]; *p* = 0.003). In contrast, the duration of ART did not significantly influence viral suppression (Table 2). Although each additional week on treatment showed a slight increase in viral suppression (AOR = 1.01, 95% CI [0.99–1.03]; *p* = 0.252) after adjusting for the age of the child, CD4 count, and drug regimen, this association was not statistically significant. Children receiving ABC/3TC/LPV-r had reduced odds of about 88% of achieving viral suppression compared with those on TDF/3TC/DTG (AOR = 0.12, 95% CI: 0.04–0.41). Children on ABC/3TC/EFV also had lower odds of suppression (AOR = 0.34), although this result did not reach statistical significance (*p* = 0.071), (Table 2).

**Table 2:** Adjusted and unadjusted logistic regression of viral load status for children on ART seen at PCOE in Lusaka, Zambia, from 1st January 2016 to 31st December 2018.

HIV viral suppression	COR	*p* value	95% CI	AOR	*p* value	95% CI
Age (years)	0.99	0.833	0.93, 1.06	0.80	0.003	0.71, 0.95
Duration on ART (weeks)	1.01	0.368	0.99, 1.02	1.01	0.252	0.99, 1.03
Weight (Kg)	1.01	0.243	0.99, 1.04	1.05	0.142	0.98, 1.12
CD4 count						
<350cells	1			1		
>350cells	1.55	0.145	0.86, 2.79	1.53	0.199	0.80, 2.94
Sex						
Male	1			1		
Female	1.05	0.867	0.61, 1.76	1.20	0.395	0.72, 2.30
ART regimen						
TDF/XTC/DTG	1			1		
TDF/XTC/EFV	0.36	0.079	0.11, 1.13	0.23	0.025	0.06, 0.83
ABC/XTC/EFV	0.56	0.254	0.21, 1.52	0.34	0.071	0.11, 1.10
ABC/XTC/LPV-r	0.26	0.004	0.10, 0.64	0.12	0.001	0.04, 0.41

COR, crude odds ratio; AOR, adjusted odds ratio; CI, confidence interval.

## 4 Discussion

Virological suppression has been used as a strong indicator for ART adherence (20). In this study, virological suppression was achieved in 78.4% of children after at least 6 months on ART, a figure that remains lower than the UNAIDS target of 95% for individuals on treatment (21). This finding is consistent with several studies from Sub-Saharan Africa, including from South Africa, Ethiopia, and Tanzania, which have similarly reported suppression rates below global targets (22–24). These variations likely reflect differences in sample characteristics, study design, and programmatic factors influencing paediatric ART adherence and outcomes (25).

A key finding in this study was the strong association between the ART regimen and virological suppression. Children receiving TDF/3TC/DTG demonstrated significantly higher rates of viral suppression compared with those on other regimens, whereas those on ABC/3TC/LPV-r, TDF/3TC/EFV, and ABC/3TC/EFV regimens had a reduced chance of viral load suppression of about 88, 77, and 66%, respectively. These findings suggest that reduced viral suppression may be driven by factors such as poor adherence and drug resistance, which are often linked to pill burden, adverse drug effects, stigma, and limited psychosocial support (26–28). LPV-r -based regimens, in particular, have been associated with poor gastrointestinal tolerability, high pill burden, and metabolic complications, all of which may negatively affect adherence and treatment outcomes (29). Previous studies have shown improved virological outcomes and fewer adverse effects with alternative protease inhibitors compared with lopinavir/ritonavir, further supporting these observations (29–31). In contrast, DTG-based regimens, which are administered once daily and are generally well tolerated, are more likely to promote adherence and sustained viral suppression among children (23).

These findings are consistent with evidence from a South African retrospective cohort study, which reported superior virological outcomes among children receiving DTG-based regimens compared with those on LPV/r-based second-line therapy (32). Furthermore, children with a baseline CD4 count above 350 cells/mm^3^ demonstrated higher odds of viral suppression, supporting the role of immune competence in effective viral control. In contrast, no significant association was observed between virological suppression and either duration on ART or time from HIV diagnosis to ART initiation, suggesting that regimen potency and patient-related factors may play a more critical role in treatment outcomes than treatment duration alone in this cohort (33, 34).

Despite this study showing that TDF/3TC/DTG and TDF/3TC/EFV which are more tolerable and taken once daily, were administered to older children older than 5 years and 10 years, an increase in age was associated with a reduced likelihood of achieving virological suppression, with each additional year corresponding to an approximately 20% decrease in the odds of suppression. This finding may reflect age-related adherence challenges, as older children are more likely to self-administer medication and may experience greater difficulty tolerating adverse drug effects. Reduced caregiver supervision, treatment fatigue, and psychosocial factors such as stigma and disclosure challenges may further compromise adherence in this age group. Similar associations between increasing age and poorer virological outcomes among children and adolescents on ART have been reported in other settings, underscoring the need for age-specific adherence support strategies, particularly for children older than five years (35).

## 5 Limitations

This study had some limitations. First, the ABC- and TDF-based regimens differed in composition and collection of information on any drug switches to newer DGT containing formulations due to virologic failure or toxicity was not done, which limited direct comparison of the individual drug effects on viral suppression. Second, the unequal distribution of participants across ART regimens may have influenced the observed associations. Third, the use of retrospectively collected routine data restricted control over data completeness and limited the inclusion of potential confounders such as adherence measures and resistance profiles. Nonetheless, the study included a relatively large sample size, and rigorous data management procedures were employed to ensure data quality. Future prospective longitudinal studies incorporating adherence and resistance testing are recommended to further elucidate determinants of virological suppression in children.

## 6 Conclusion

This study found that 78.4% of children receiving ART achieved virological suppression, a level that remains below the UNAIDS target of 95%. ART regimen and age of the child emerged as the primary predictors of viral load suppression. Among the regimens evaluated, TDF/3TC/DTG was associated with the highest likelihood of virological suppression, whereas ABC-based and EFV- or LPV-r–based regimens demonstrated lower odds of achieving suppression. In addition, increasing age was significantly associated with reduced viral suppression, highlighting the vulnerability of older children to adherence-related challenges.

These findings underscore the importance of optimizing ART regimens, particularly expanding access to DTG-based combinations, and strengthening age-specific adherence support interventions for children, especially those older than five years. Future prospective longitudinal studies with balanced regimen distribution and inclusion of adherence and resistance measures are recommended to further inform evidence-based paediatric ART strategies in Zambia and similar settings.

## Data Availability

The original contributions presented in the study are included in the article/Supplementary material, further inquiries can be directed to the corresponding author.
