# Supplementary material for: HIV viral suppression outcomes of Tenofovir- and Abacavir-based antiretroviral regimens among children on ART in Zambia
Source: Front Public Health. 2026 Jul 8;14:1860427. doi: 10.3389/fpubh.2026.1860427 (PMC13388562; doi:10.3389/fpubh.2026.1860427)
Supplement: Supplementary file 1 [file Data_Sheet_1.PDF]

Appendix: Data extraction tool

ID Number……………………Date…………Investigator……………………………

Section A

	**Characteristic**	**Possible options**	**Tick/indicate**
1	Sex	1= Female	
		2= Male	
2	Age	1=18 months−4 years	
		2=5 years−9 years	
		3=10 years−14 years	
3	Viral load	0=Less than 1000 copies/ml	
		1=More than 1000 copies/ml	
4	Time on ART (Weeks)	Continuous values	
5	Weight of child (Kg)	Continuous	
6	Time lag diagnosis and treatment (weeks)	Continuous values	
7	ART regimen child is taking	1=NRTI+NNRTI	
		2=NRTI+PI	
		3=NRTI+INSTI	
8	CD_4 count at initiation	1= <350 cells	
		2= ≥350 cells	
